# Dielectric Properties of Ovine Heart at Microwave Frequencies

**DOI:** 10.3390/diagnostics11030531

**Published:** 2021-03-16

**Authors:** Niko Ištuk, Emily Porter, Declan O’Loughlin, Barry McDermott, Adam Santorelli, Soroush Abedi, Nadine Joachimowicz, Hélène Roussel, Martin O’Halloran

**Affiliations:** 1 Translational Medical Device Laboratory, National University of Ireland Galway, Costello Road, H91 TK33 Galway, Ireland; barryjames.mcdermott@nuigalway.ie (B.M.); martin.ohalloran@nuigalway.ie (M.O.); 2 Department of Electrical and Computer Engineering, The University of Texas at Austin, Austin, TX 78712, USA; emily.e.porter@ieee.org (E.P.); adam.m.b.santorelli@gmail.com (A.S.); 3 Department of Electronic and Electrical Engineering, Trinity College Dublin, College Green, D02 PN40 Dublin 2, Ireland; d.oloughlin@tcd.ie; 4 Sorbonne Université, CNRS, Laboratoire de Génie Electrique et Electronique de Paris, 75252 Paris, France; Soroush.abedi@upmc.fr (S.A.); nadine.joachimowicz@paris7.jussieu.fr (N.J.); helene.roussel@sorbonne-universite.fr (H.R.); 5 Université Paris-Saclay, CentraleSupélec, CNRS, Laboratoire de Génie Electrique et Electronique de Paris, 91192 Gif-sur-Yvette, France

**Keywords:** heart, dielectric properties, biological tissues, atrial fibrillation, electromagnetic heating, ablation

## Abstract

Accurate knowledge of the dielectric properties of biological tissues is important in dosimetry studies and for medical diagnostic, monitoring and therapeutic technologies. In particular, the dielectric properties of the heart are used in numerical simulations of radiofrequency and microwave heart ablation. In one recent study, it was demonstrated that the dielectric properties of different components of the heart can vary considerably, contrary to previous literature that treated the heart as a homogeneous organ with measurements that ignored the anatomical location. Therefore, in this study, we record and report the dielectric properties of the heart as a heterogeneous organ. We measured the dielectric properties at different locations inside and outside of the heart over the 500 MHz to 20 GHz frequency range. Different parts of the heart were identified based on the anatomy of the heart and their function; they include the epicardium, endocardium, myocardium, exterior and interior surfaces of atrial appendage, and the luminal surface of the great vessels. The measured dielectric properties for each part of the heart are reported at both a single frequency (2.4 GHz), which is of interest in microwave medical applications, and as parameters of a broadband Debye model. The results show that in terms of dielectric properties, different parts of the heart should not be considered the same, with more than 25% difference in dielectric properties between some parts. The specific Debye models and single frequency dielectric properties from this study can be used to develop more detailed models of the heart to be used in electromagnetic modeling.

## 1. Introduction

Knowledge of the dielectric properties (relative permittivity, $\varepsilon_{r}$, and conductivity, σ) of biological tissues is important in the design and optimization of novel diagnostic and therapeutic technologies such as electrical impedance tomography, microwave imaging, hyperthermia, radiofrequency (RF) ablation, and microwave (MW) ablation systems [1]. Modeling of the thermal effects of the electromagnetic (EM) field and organs in the human body requires the knowledge of the geometry of the organ, and tissue heat transfer mechanisms within and surrounding the organ as well [2].

Specifically, while MW cardiac ablation has shown promise in treatment of atrial fibrillation (AF), the technology faces challenges such as the heat sink effect, difficulties in getting reliable energy transmission between the microwave applicator and the functioning heart, and in avoiding collateral tissue damage from stray or fringe fields around the applicator [3,4,5,6]. The development of clinical protocols for treatment planning is strictly linked to the availability of numerical models able to accurately predict the procedure outcomes [7]. Parameters such as blood flow, dielectric and thermal properties of the tissues all need to be incorporated in the development of accurate numerical models [3,4,5,6]. Precise knowledge of tissue dielectric properties can facilitate a more accurate simulation of device performance [8] as penetration of a microwave field into a tissue medium is dependent on the dielectric properties of the tissue [6]. This can help optimize device geometry and energy delivery parameters [8]. The heterogeneity within the heart should be considered in order to achieve an accurate dielectric profile of the organ [9].

The dielectric properties of biological tissues can be accessed from comprehensive online databases including the Italian National Research Council database [10] and the IT’IS Foundation database [11]. Both databases are based on the parametric models developed by Gabriel et al. in 1996 [12]. The data collected and measured by Gabriel et al. are to this day accepted as standard for dielectric properties of human tissues including the heart [9], despite the improvement in the measurement protocols in the years since the study.

Previous studies have reported on the dielectric properties of the heart. The Gabriel et al. parametric model for the heart tissue is based on the data gathered from the literature [13] as well as data from their experimental study [14]. In their experimental study, Gabriel et al. measured dielectric properties ex vivo on excised animal tissue and human autopsy material. The dielectric properties of the heart were measured ex vivo on human tissues obtained from an autopsy performed between 24 and 48 h after death at frequencies between 300 kHz to 20 GHz, and on ovine heart at frequencies between 10 Hz and 20 GHz, within 2 h of death. Both the human and ovine heart tissues were measured at 37 °C. Part of the data gathered from the literature that was measured over the microwave frequency range includes data from in vivo experiments on bullfrog [15] and in vitro experiments on canine tissue [16].

In their study from 1987, Xu et al. measured canine tissues in vitro [16]. The tissues were immersed in saline for up to 12 h, which is not considered best practice by today’s standards since such immersion can modify the dielectric properties [1]. While the room temperature is reported, the temperature of the tissue is not.

In a more recent study by Fornes-Leal et al. from 2019 [17], dielectric properties of the porcine heart, among other porcine thoracic tissues, were measured in vivo at the frequency range from 500 MHz to 26.5 GHz. In this study, the heart was treated as a homogeneous organ and while the measurements were made at five different locations on each organ that was measured, the anatomical part of the heart that was measured is not specified.

However, further work is needed to accurately characterize the dielectric properties of the heart. Challenges include the inherent variation in all tissues (both within patient and between patients) [18], as well as the anatomical and functional heterogeneity of the organ [19,20]. Indeed in a recent study, Salahuddin et al. experimentally demonstrated that treating the heart as a homogeneous organ may not provide an accurate dielectric representation of the entire organ [21].

Hence in this study, the heart was considered a heterogeneous organ with care taken to discretely measure the dielectric properties of distinct locations on both the heart and great vessels. These locations were identified based on an anatomical and functional categorization of the heart and vessels. Dielectric data were collated according to the part of the heart that measurement location corresponds to. Measurements on different parts of the heart were taken in order to achieve a full dielectric profile of the heart. The study by Salahuddin et al. [21] considered only tissues on the outside of the heart, while in this study we measured both the interior and the exterior of the heart. The level of detail in terms of the number of measurements on a specific location on the heart, and the number of parts of the heart considered in this study exceeds that of any previous study.

For each part of the heart, we provide the fitted Debye parameters that can be used in broadband simulations from 500 MHz to 20 GHz, as well as single frequency dielectric properties at the most commonly used frequency for microwave ablation (2.4 GHz) [6].

## 2. Background

The heart is the center of the circulatory system, and acts as a dual muscular pump. On the right side of the heart, carbon dioxide rich blood from the vena cavae enters the right atrium before being pumped out from the right ventricle to the lungs through the pulmonary artery [19]. Oxygen rich blood from the lungs returns through the pulmonary vein to the left atrium, before being pumped out from the left ventricle to the entire body via the aorta. From a histological perspective, the parts of the heart are arranged to fit a certain function. The endocardium is the inner lining of the heart, continuous with the endothelium of blood vessels [20]. Inside this layer is the cardiac muscle layer called the myocardium, which is thicker on the left side due to the need for greater force in pumping blood to the entire body as opposed to the right side that serves the lungs [16]. On the exterior surface of the heart is the epicardium, a serous membrane. Finally, a layer of fibrocartilage separates the atria and ventricles supporting the heart valves and forming openings between the chambers [20].

A number of pathologies can affect the heart. AF is the most common heart arrhythmia and is a major global health burden worldwide [22,23,24]. In AF the atria contracts rapidly and irregularly, which causes an inadequate pumping of the blood from the atria to the ventricles. AF is usually not immediately life threatening; however, it is still one of the major causes of stroke, heart failure, sudden death, and cardiovascular morbidity [25]. Despite these facts, the optimal management of AF remains unclear [26]. AF can be treated using various ablation modalities such as RF and MW catheter ablation and hence accurate knowledge of dielectric properties of the heart is important.

Catheter ablation of AF is a well-established rhythm control option for symptomatic paroxysmal, persistent and probably long-standing persistent AF [25,27]. It is generally a second-line treatment after failure of or intolerance to antiarrhythmic drug therapy [25,27,28] and it has developed from a specialized, experimental procedure into a common treatment to prevent recurrent AF [25,29].

The most recent guidelines from the European Society of Cardiology from 2016 recommend three core strategies to AF management [25]. These strategies include stroke prevention, rhythm control, and rate control therapies [25], with initial therapy being directed toward the rhythm control [30,31].

Catheter ablation is a treatment approach where a well-localized region of the endocardial tissue mediating the arrhythmias is destroyed via energy applied through a catheter, which is inserted into the heart through a vein [32]. Depending on the type of energy source used there are different types of ablation: radiofrequency (RF), microwave (MW), ultrasound, laser and cryoablation [33]. RF and MW ablation are electromagnetic (EM) based and they require the knowledge of the dielectric properties to calculate the Specific Absorption Rate (SAR) for treatment planning [9,34,35]. Typical frequency of RF ablation is in the range of 350 kHz to 700 kHz (usually 500 kHz on commercially available RF generators) [36], while the most commonly used frequencies for MW ablation are 915 MHz and 2.45 GHz [6].

RF ablation has become widely accepted treatment for most atrial and ventricular arrhythmias, including AF [33]. It relies on electrical conduction through the tissue, where RF current is able to pass through the tissue because of the abundance of ionic fluid present in the tissue and since the tissue is not a perfect conductor, RF current causes resistive heating (the Joule effect) [37]. The mechanism by which RF current heats tissue is resistive heating of a narrow rim (<1 mm) of tissue that is in direct contact with the ablation electrode. Deeper tissue heating occurs as a result of passive heat conduction from this small region of volume heating [38].

MW ablation is a special case of dielectric heating in which an alternating EM field is applied to an imperfect dielectric material. In tissue, heating occurs because the EM field forces polar molecules like water in the tissue to oscillate and produce heat [8]. By inducing dielectric losses in polar molecules it may be theoretically possible for a greater amount of tissue to be heated as compared to that heated directly by RF current [33,38]. With MW ablation it is possible to rapidly create long linear lesions at anatomically complex sites [25]. Early clinical and preclinical data support the use of MW ablation for the treatment of AF [6]. Since MW ablation is based on dielectric heating, improved knowledge of dielectric properties of the tissues within the heart will improve the efficacy of MW ablation of different heart tissues.

## 3. Materials and Methods

### 3.1. Measurement Method

In line with best practices for dielectric characterization of biological tissues, all measurements in this work were collected using the open-ended coaxial probe method. The method is simple, requires minimum sample handling and is non-destructive. The slim form open-ended coaxial probes from 85070E Dielectric Probe Kit (Keysight, Santa Rosa, CA, USA) that were used in this study are suitable for both ex vivo and in vivo measurements over the frequency range of interest [1,39,40]. While this study considers ex vivo measurements, the method has been applied successfully to in vivo measurements [1,17,40]. While care needs to be taken to control for confounding factors such as the temperature of the tissue, time from excision and measurement uncertainties, the open-ended coaxial probe method has been demonstrated to be the most applicable for measuring the dielectric properties of biological tissues over the microwave frequency range [1].

The dielectric properties and related metadata were collected following the MINDER guidelines for reporting of dielectric data of biological tissues [41]. Following the FAIR principles [42], the dielectric properties data and metadata are available online [43].

Two vector network analyzers (VNA) were used to record the $S_{11}$ parameters: E8362B PNA (Agilent, now Keysight, Santa Rosa, CA, USA) (VNA1) and 5063A ENA (Keysight, Santa Rosa, CA, USA) (VNA2). We used two VNAs to increase the throughput by simultaneously conducting measurements on two heart samples. This helped minimize the time between the excisions and the measurements.

**Figure 1:**
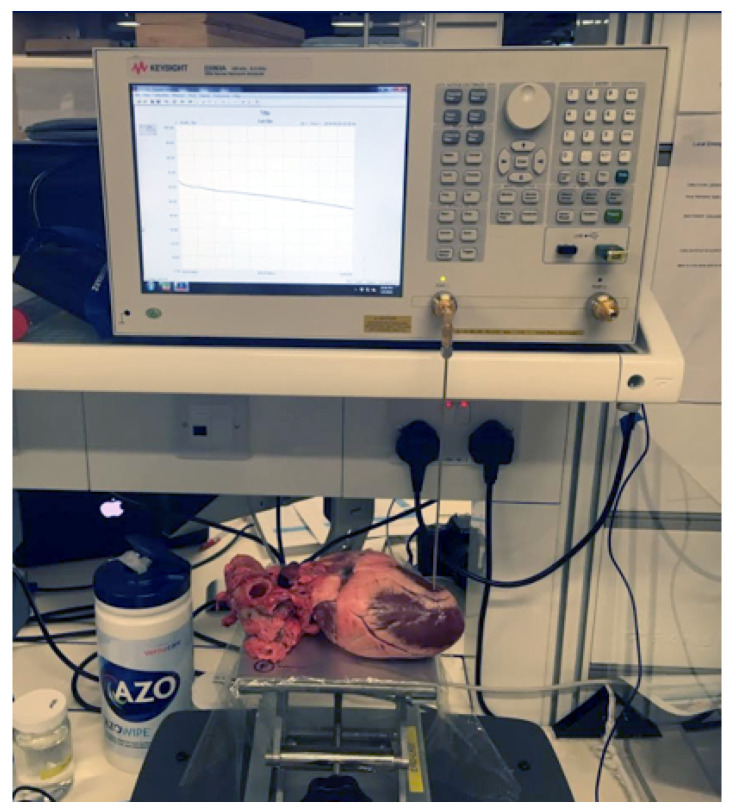
Measurement setup showing VNA2 directly connected to the slim form probe. The sample is brought in contact with the probe by lifting the table while making sure we do not apply excessive probe-sample pressure. The exclusion of the cable from the setup eliminates one source of measurement uncertainty.

Both VNAs were set to record the $S_{11}$ parameters at 101 discrete frequency points. The frequency points were distributed logarithmically over the frequency range from 200 MHz to 20 GHz for measurements performed with VNA1 and over the frequency range from 200 MHz to 8.5 GHz for VNA2. The two frequency ranges were determined by the respective VNA operating frequencies and they both cover the most commonly used frequencies for MW ablation (915 MHz and 2.45 GHz [6]). Open-ended coaxial probes operating frequency range starts at 500 MHz [38], so the results were filtered to exclude the measurement at lower frequencies. Therefore the final frequency range was 500 MHz–20 GHz in the case of VNA1 (81 points) and 500 MHz–8.5 GHz in the case of VNA2 (77 points). The logarithmic distribution of frequency points results in more accurate modeling of the measured data, especially at lower frequencies [44]. To eliminate noise from cable movement and reduce measurement uncertainty, we used two slim form open-ended coaxial probes from 85070E Dielectric Probe Kit (Keysight, Santa Rosa, CA, USA), connected to the VNAs using a right-angle SMA connector. The measurement setup with an open-ended coaxial probe connected to VNA2 can be seen in Figure 1. To reduce movement of the measurement setup, tissues were placed on top of the lift table and brought in contact with the probe. While the manufacturer recommends the probe should be inserted 5 mm into the material under test, this was avoided in this study as it would cause damage to the tissue. Instead, a good contact between the tissue and the probe has been considered to be adequate in numerous studies on dielectric properties of biological tissues [1,17,18,21,45,46]. The pressure applied between the probe and the sample was moderate as excessive probe-sample pressure can cause inaccurate measurements [1]. The measured $S_{11}$ parameters were converted to the dielectric properties using the N1500A Materials Measurement Suite (Keysight, Santa Rosa, CA, USA).

### 3.2. Calibration and Validation

The measured $S_{11}$ parameters suffer from the instrument’s systematic (repeatable) errors [47]. We used the one-port calibration method to correct this type of error. The calibration was performed before the first measurement on each heart sample and was repeated approximately every 35 min. We used open circuit, short circuit, and deionized water (DIW) as the three standard loads in the calibration procedure. The temperature of DIW was measured for every calibration using a digital thermometer and it ranged from 23.5 °C to 26.5 °C.

The validation of the calibration was performed after each calibration and each time the part of the heart that was being measured was changed. The validation is conducted by measuring the dielectric properties of a material with a known dielectric model and then comparing the measured data to the model. The material is usually a standard liquid, such as deionized water, saline solution, or methanol. Ideally, the measurements should match the model perfectly and any error between the measurements and the model is considered the validation error.

In this study, we used 0.9% sodium chloride (NaCl) in aqueous solution. We measured the dielectric properties and temperature of sodium chloride solution. The temperature was measured with the digital thermometer for each validation and it ranged from 23.6 °C to 25.7 °C. The dielectric model for the NaCl solution was obtained from the literature [48] and was calculated for the given concentration and temperature over the desired frequency range. The mean error over all frequencies between the results of the validation measurement and values obtained from the model was considered the validation error for that particular validation measurement. The validation error was calculated separately for the relative permittivity and conductivity. The mean and maximum validation errors across *N* = 99 validations are reported in Table 1. The difference between the validation measurement and the model was on average 1.36% in relative permittivity and 1.78% in conductivity.

### 3.3. Heart Samples and Measurements

The thoracic contents from four sheep (*n* = 4, labeled A1–A4) were collected from an abattoir immediately after humane euthanasia using a captive bolt and carotid artery dissection. No living animal was used for the sake of the experiments. All animal tissues were sourced from a commercial abattoir operating in full compliance with Irish and EU law and the study was conducted in compliance with the ethical guidelines at NUI Galway. All work related to animals complied with the 3Rs principles for humane research (replace, reduce, refine) where using ex vivo tissues instead of living animals is considered one of the replacement methods [49]. Once collected, the excised thoracic contents of the animals were placed in an airtight container in order to limit the dehydration of the heart tissue. The hearts were dissected from the rest of the thoracic contents right before the beginning of the measurements.

The animal blood was drained by dissecting their carotid artery. Since heart is a highly perfused organ, this was possibly an issue. However, Farrugia et al. concluded that the changes in the in vivo and ex vivo dielectric properties cannot be associated with blood perfusion [45]. Their conclusion comes from measurements on liver, which is a highly perfused organ. While heart is considered to be a highly perfused organ as well, in this study we measured the dielectric properties of heart muscle and vessel tissues, which are not so highly perfused.

The times of excision of the thoracic content (approximately the same as the time of death) were recorded, as well as the time of each measurement. The time from the excision of thoracic content to the first measurements was approximately 4 h and the time from the excision to the last measurement was approximately 8.5 h. The time of the dissection of the heart from the thoracic content is the same as the time of the first measurement.

**Figure 2:**
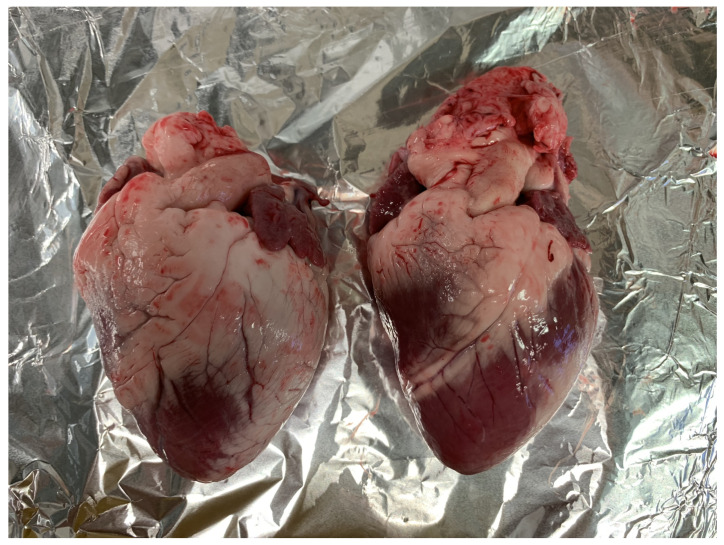
Two heart samples from A1 (left) and A2 (right). The hearts are different in size, shape and the amount of pericardial fat covering the epicardium. The pericardial fat extends around the whole heart presenting a challenge when measuring the dielectric properties of the tissues on the exterior surface.

Figure 2 shows two hearts (Left: A1, Right: A2) illustrating the differences in size, shape and pericardial fat. A2 (on the right), has noticeably less fat. Parts of the epicardium are covered with a layer of fat that is so thin it is almost transparent and care has to be taken to avoid measuring the combination epicardium with fat. Dielectric measurements can show a high sensitivity to even thin layers of fat [46].

We identified nineteen distinct locations based on the heart anatomy and function. Fifteen measurements (*n* = 15) were conducted around each location, moving the probe slightly each time, in order to ensure that we have repeatable measurements. The measurements performed on two locations, namely the mitral valve and tricuspid valve, did not produce consistent results and therefore they were omitted. The measurement results from the remaining seventeen locations were grouped into six groups according to the part of the heart they belong to: epicardium, myocardium, endocardium, the exterior surface of the atrial appendage, the interior surface of the atrial appendage and the luminal surface of the great vessels. The total number of measurements on a specific part of the heart is the number of repeated measurements on each measurement location (*n* = 15) multiplied by the number of locations that correspond to that part of the heart.

After the measurements on the exterior of the heart sample were completed, we performed a series of transverse sections of the heart in order to access the endocardium of the ventricles. This sectioning also exposed the myocardium. We performed fifteen measurements on the interior surface of the left atrium and fifteen measurements on the interior surface of the right atrium. These thirty measurements (*n* = 30) constitute half of the measurements performed on the endocardium. The dielectric properties of myocardium were measured on the septum of the heart sample (*n* = 15 measurements).

Next, we performed further transverse sections at the level of the atria and performed fifteen measurements on the endocardium of the left atria and fifteen measurements on the endocardium of the right atria. These thirty measurement (*n* = 30) constitute the other half of the measurements performed on the endocardium, totaling sixty measurements (*n* = 60). The measurements on the vessels were performed on the luminal surface of the four great vessels of the heart: the aorta, pulmonary artery, pulmonary vein, and the vena cava (*n* = 60). The luminal surfaces were accessed by using longitudinal cuts across vessel walls.

This procedure was repeated for each of the four heart samples, giving a total of *n* = 1020 measurements with seventeen locations on the four heart samples measured fifteen times each.

**Figure 3:**
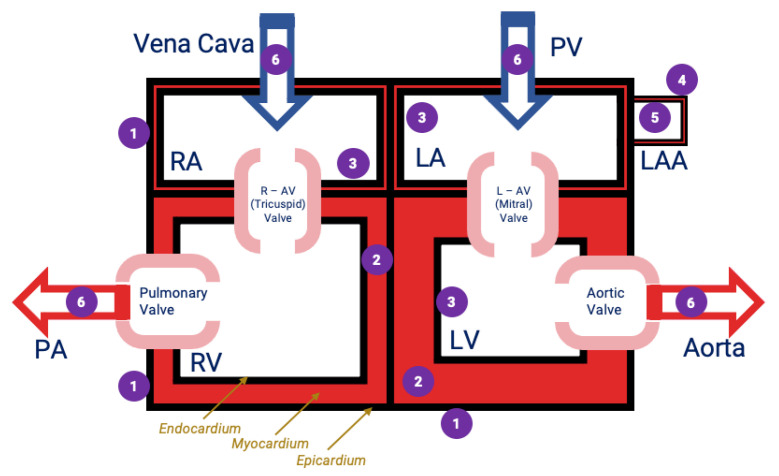
Schematic of measurement locations. *n* = 17 distinct locations were selected with 15 measurements at each location. These locations were divided into 6 groups as (1) epicardium; (2) endocardium, (3) endocardium, (4) the exterior surface of the atrial appendage, (5) the interior surface of the atrial appendage and (6) luminal surface of the great vessels. These groupings 1–6 are shown in the figure. (RA = right atrium, LA = left atrium, RV = right ventricle, LV = left ventricle, R–AV Valve = right atrioventricular valve, L–AV Valve = left atrioventricular valve, PA = pulmonary artery, PV = pulmonary vein, LAA = left atrial appendage).

Figure 3 provides a schematic of the seventeen measurement locations, each a member of one of the six groups based on the location and nature of the cardiac tissue at that location.

We recorded the temperature of the tissue at the point of contact with the probe at the time of the first and the last measurement on that particular location. We used the Fluke 62 MAX+ (Fluke, Everett, WA, USA) infrared thermometer to measure the temperature. The minimum temperature of the tissue measured was 23.7 °C and the maximum temperature was 25.6 °C. The temperature of the room was controlled during the measurements at 25 °C.

### 3.4. Fitting Model

To facilitate the incorporation of the dielectric data in the simulation of exposure situations and the calculation of internal fields within the body, it is convenient to express their frequency dependence as parametric expressions to provide access to data at all frequencies of interest [12].

The two most commonly used parametric models of dielectric properties are the Cole–Cole model and Debye model [50,51]. The Debye model can easily be expressed in both time and frequency domains and is not computationally expensive, which makes it widely used in electromagnetic numerical simulations [50]. The Debye model is defined as:

(1) $$\hat{\varepsilon }=\varepsilon_{\infty }+\sum_{p=1}^{n}\frac{\Delta \varepsilon_{p}}{1+j\omega \tau_{p}}+\frac{\sigma_{s}}{j\omega \varepsilon_{0}},$$

where $\varepsilon_{\infty }$ is the permittivity at frequencies where $\omega \tau_{p}\gg 1$, $\sigma_{s}$ is the static ionic conductivity, $\varepsilon_{p}$ is the magnitude of *p*th dispersion, $\tau_{p}$ is the relaxation constant of *p*th dispersion, ω is angular frequency and $\varepsilon_{0}$ is the permittivity of free space.

In this study, we fitted the mean of the measurements of all measured parts of the heart for each of the four heart samples to a three pole Debye model. In this study, we used the least squares method [52], which is the most common approach to obtain the best parameters of a model [12]. We calculated the fit error between the mean of the measured data and the resulting Debye model for each part of the heart.

### 3.5. Statistics

The results for relative permittivity and conductivity at single frequency (2.4 GHz) are presented with mean and standard deviation (SD). One-way analysis of variance test (one-way ANOVA) was used to compare the mean permittivity and mean conductivity values for each heart sample (total number of *n* = 8 tests). A *p* value of < 0.05 was accepted as statistically significant.

## 4. Results

### 4.1. Dielectric Properties of the Heart

**Figure 4:**
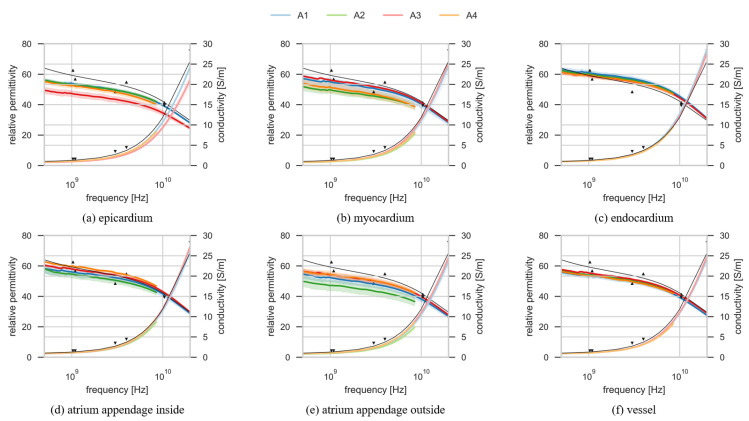
Dielectric properties of six parts of the heart (**a**–**f**). Each plot shows relative permittivity and conductivity for one part of the heart, measured on four hearts. Four colors correspond to four hearts: A1 is blue, A2 is green, A3 is red and A4 is orange. Darker lines are relative permittivity and lighter lines are conductivity. The measurement results plotted with the green and the orange lines are measured with the E5063A VNA. The measurement results plotted with the blue and red line are representing the measurements performed with the E8362B VNA. Each line is plotted with the corresponding mean ± 2 standard deviations confidence interval. The thin black lines are the model for the relative permittivity and conductivity of the heart muscle from the literature [10,11,12,51]. The model is based on the data from experimental studies on several different species. The triangle markers are the data points from measurements on human tissues [14].

Figure 4 shows the results of the dielectric property measurements performed on four ovine heart samples. Each subplot shows the results of the measurements on one of the six different parts of the heart examined in this study. Each subplot shows the results of the measured dielectric properties on four ovine hearts. Blue lines correspond to measurements from A1, green lines from A2, red lines from A3 and orange from A4. Further, each heart is represented with different shades of the respective color, with the darker shades representing relative permittivity and the lighter shades representing conductivity. All the measurement results are plotted as the mean of individual measurements on that part of the heart, with shaded area around the mean that represents the mean ± two standard deviations confidence interval. The average (±standard deviation) temperature of the tissues during the measurements was 23.5 °C (±0.9 °C). For the comparison of the measurement results with the data from the literature, the thin black line represents the model from Gabriel et al. [10,11,51]. The markers are the data from Gabriel et al. for human tissue at 37 °C.

The differences between the measured values can be due to several reasons. In this study the data were measured on ovine heart while Gabriel et al. based their model on the data from literature and data from experimental studies on several different species, as was discussed in Section 1 [12]. Considering just the human data from Gabriel et al., at least four factors could explain differences between the data measured by Gabriel et al. and the experimental results in this study: the temperature of the tissue, the level of hydration, inter-species variability and variability between different parts of the organ.

Firstly, the data in this paper were acquired at room temperature (between 21.7 and 25.6 °C), whereas all measurements reported by Gabriel et al. were acquired at body temperature (37 °C) and how this temperature is achieved is not reported [51]. Although the dielectric properties are known to change with temperature and the dielectric properties at body temperature are desired, we decided to avoid heating the tissue as it would affect tissue dehydration and the measurements would not be representative. Instead, in this work, the tissues are not heated and the measurement temperature is reported in line with the MINDER principles. The downside of this approach is that the dielectric properties are temperature dependent [53,54,55,56]. Therefore, the results from this study are not an accurate representation of dielectric properties of heart at body temperature but rather at room temperature.

Secondly, it is possible that there is an inter-species variation in the dielectric properties of the heart. However, the differences in the dielectric properties between animal and human species are not systematic and the variation in tissue properties within a species may well exceed variations between species [13]. Thirdly, in our study the time from excision was approximately 4 h and the effect of different times from excision to measurements were considered. In a study by Gabriel et al., human material was obtained 24 to 48 h after death [14], which is a much longer period of time than in this study. Fourthly, the last factor that explains the difference between the results is the variability between the different parts of the heart, which is specifically examined in this study.

**Figure 5:**
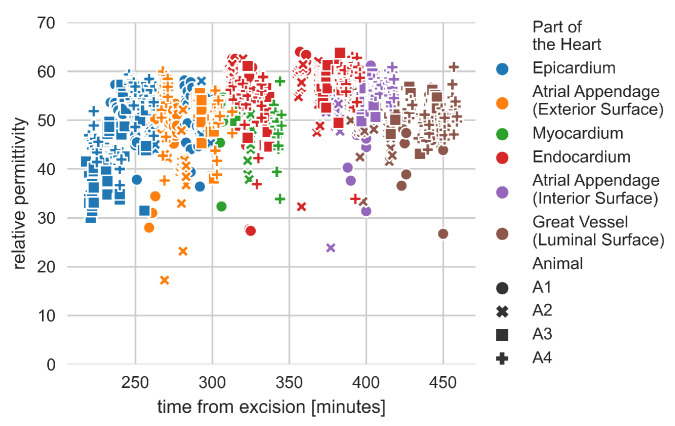
Single frequency (2.4 GHz) relative permittivity measurements versus time from excision in minutes. Different marker colors represent different parts of the heart. Different marker shapes represent measurements on different hearts. There was no obvious trend observed in changes of dielectric properties measured with respect to time from excision.

Figure 5 displays all measurements of relative permittivity at 2.4 GHz. The measurements are plotted versus time from excision in minutes. Figure 5 serves to illustrate whether the dehydration of the tissue, which to some degree is inevitable over time, played a significant role as a confounding factor during the measurement process. If this were the case then we would expect to see a trend such as decrease of relative permittivity over time as a consequence of the tissue dehydration [57]. There was no obvious trend observed in changes of dielectric properties measured with respect to time from excision. All of the data points are well clustered together with limited outliers.

Table 2 shows the relative permittivity and conductivity for all parts of the heart of all four ovine heart samples at 2.4 GHz, suitable for modeling ablative technologies, which often operate at 2.45 GHz [36]. These results are suitable in single frequency numerical simulations, where each part of the heart dielectric properties can be set using single frequency values for relative permittivity and conductivity. The values are given as the mean of all measurements for each part of each of the hearts. The values for relative permittivity range from 44.18 for the epicardium of A3, to 57.83 for the endocardium of A1, which is more than 26% difference. The conductivity values range from 1.64 S/m measured on the exterior surface of the appendage of A2, to 2.10 S/m, as measured on the endocardium of A1, which is more than 25% difference. All the values in the table are given with their standard deviation. The standard deviation in relative permittivity was between 1.13 and 9.40 and in conductivity between 0.04 and 0.35 S/m. The one-way ANOVA tests null-hypothesis is that the mean values are the same. The hypothesis is rejected with *p* < 0.001 for all tests.

### 4.2. Debye Models

Parameters of the three-pole Debye model fitted to the mean of the measurement data are presented in Table 3. Each part of the heart is represented with four sets of model parameters that correspond to the measurements performed on four ovine heart samples. These sets of parameters are suitable for use in the broadband simulation scenarios, over the frequency range that the models were fitted and verified over.

The models were verified by calculating the mean difference in percentage across all frequency points, between the mean of the measurement data and the model. This mean difference is the error of the fit, shown in Table 4, and in the worst case it was 0.51% for relative permittivity and 0.75% for conductivity. The low values of the fitting errors are indicating that there is a good fit between the measurement data and the model.

## 5. Conclusions

In this study, we performed, to our best knowledge, the most detailed dielectric characterization of the heart in the microwave frequency range. In all prior studies, except for that of Salahuddin et al. [21], the heart was considered a homogeneous organ, with only one value or one model being reported for the whole organ. The study of Salahuddin et al. demonstrated that a heterogeneous treatment of the heart may lead to a more accurate dielectric characterization of this important organ.

This present study is the first to report dielectric properties and parameters required for parametric models for different parts of the heart. Specifically, six parts of the heart measured at seventeen locations were considered. The results show that two different parts of the heart can differ by more than 25% in both relative permittivity and conductivity, as measured at a single frequency relevant in microwave thermal ablation (2.4 GHz). The broadband dielectric properties are reported as parameters of the three-pole Debye model. Four models were fitted for each part of the heart that was covered in this study.

The results for single frequency dielectric properties and the parameters of the Debye models can be used to build an anatomically correct model of the heart with fully accurate dielectric profile. These models are suitable for numerical modeling of the interaction between electromagnetic fields and the heart at microwave frequencies, allowing for improved accuracy, and ultimately better patient outcomes, of cardiac microwave ablation treatment.

## Figures and Tables

**Table 1:** The mean and the maximum value of the validation error across all frequency points, in percentage. The mean error is the mean across all validation measurements (*n* = 99). The maximum error is the maximum across all validation measurements.

	Mean Error (%)	Maximum Error (%)
Relative permittivity	1.36	4.92
Conductivity	1.78	3.64

**Table 2:** The values of the relative permittivity and conductivity of the measured parts of the heart at 2.4 GHz. The values given as the mean and standard deviation (SD) across all measurements on a particular part of the heart. These part of the heart specific values can be used in single frequency numerical simulations.

Animal	Part of the Heart	$\varepsilon_{r}$	SD ($\varepsilon_{r}$)	σ (S/m)	SD (σ) (S/m)
A1		53.23	6.80	1.94	0.28
A2	Atrial Appendage	51.51	5.94	1.87	0.22
A3	(Interior Surface)	54.65	2.54	1.98	0.10
A4		56.68	1.98	2.03	0.09
A1		48.84	6.50	1.86	0.27
A2	Atrial Appendage	44.56	9.40	1.64	0.35
A3	(Exterior Surface)	51.11	4.11	1.93	0.15
A4		51.19	5.74	1.91	0.24
A1		57.83	6.73	2.10	0.28
A2	Endocardium	57.03	4.42	2.02	0.18
A3		56.01	4.38	2.04	0.18
A4		56.14	5.31	1.99	0.23
A1		50.41	4.39	1.90	0.17
A2	Epicardium	50.69	3.91	1.80	0.12
A3		44.18	8.22	1.68	0.29
A4		49.31	6.28	1.83	0.17
A1		51.38	5.60	1.87	0.23
A2	Myocardium	46.40	4.22	1.67	0.17
A3		52.89	1.13	1.90	0.04
A4		47.76	5.54	1.81	0.19
A1		50.79	5.29	1.88	0.20
A2	Great Vessels	51.10	4.55	1.85	0.17
A3	(Luminal Surface)	52.00	2.77	1.93	0.09
A4		50.73	3.08	1.83	0.10

**Table 3:** Three-pole Debye model parameters fitted to the mean permittivity of measurements on different parts of the heart. Each part was measured on four different hearts and is therefore represented by four sets of Debye model parameters. The model parameters for A1 and A3 are verified at frequencies from 500 MHz to 20 GHz. The parameters for A2 and A4 are verified at frequency range from 500 MHz to 8.5 GHz. These models are suitable for use in broadband numerical simulation scenarios over their respective frequency ranges.

Animal	Part of the Heart	$\varepsilon_{\infty }$	$\sigma_{s}$ (S/m)	$\Delta \varepsilon_{1}$	$\tau_{1}$ (ns)	$\Delta \varepsilon_{2}$	$\tau_{2}$ (ns)	$\Delta \varepsilon_{3}$	$\tau_{3}$ (ns)
A1		6.86	0.741	7.23	0.34	4.79	$4.01\times 10^{-2}$	43.27	$8.05\times 10^{-3}$
A2	Atrial Appendage	13.02	0.263	137.91	2.15	4.32	$1.01\times 10^{-1}$	37.74	$1.01\times 10^{-2}$
A3	(Interior Surface)	7.12	0.426	88.09	1.77	4.84	$7.89\times 10^{-2}$	46.33	$8.38\times 10^{-3}$
A4		12.71	0.210	244.73	3.03	4.59	$8.59\times 10^{-2}$	42.98	$9.64\times 10^{-3}$
A1		7.00	0.373	72.86	1.39	4.95	$6.10\times 10^{-2}$	39.72	$8.20\times 10^{-3}$
A2	Atrial Appendage	11.60	0.323	96.24	2.02	4.30	$8.44\times 10^{-2}$	31.89	$9.96\times 10^{-3}$
A3	(Exterior Surface)	7.48	0.690	11.34	0.42	4.78	$4.17\times 10^{-2}$	40.64	$8.16\times 10^{-3}$
A4		14.40	0.704	19.93	0.97	3.97	$8.51\times 10^{-2}$	36.31	$1.07\times 10^{-2}$
A1		7.53	0.124	311.85	3.41	4.70	$6.24\times 10^{-2}$	48.61	$8.41\times 10^{-3}$
A2	Endocardium	13.38	0.626	44.30	1.31	3.51	$9.77\times 10^{-2}$	43.37	$1.00\times 10^{-2}$
A3		7.36	0.734	17.80	0.74	4.36	$6.57\times 10^{-2}$	47.12	$8.20\times 10^{-3}$
A4		14.22	0.433	170.62	3.31	3.97	$1.06\times 10^{-1}$	41.88	$1.02\times 10^{-2}$
A1		7.70	0.439	63.45	1.40	5.13	$6.42\times 10^{-2}$	40.64	$8.21\times 10^{-3}$
A2	Epicardium	12.71	0.154	254.62	3.34	4.40	$8.30\times 10^{-2}$	36.84	$9.62\times 10^{-3}$
A3		6.74	0.550	14.43	0.54	4.49	$4.97\times 10^{-2}$	34.95	$8.06\times 10^{-3}$
A4		14.75	0.141	207.25	2.64	4.27	$7.95\times 10^{-2}$	33.65	$1.11\times 10^{-2}$
A1		8.34	0.538	25.33	0.86	4.73	$6.90\times 10^{-2}$	41.52	$8.54\times 10^{-3}$
A2	Myocardium	10.49	0.035	396.00	5.37	4.81	$1.03\times 10^{-1}$	35.23	$9.50\times 10^{-3}$
A3		8.12	0.533	26.58	0.92	5.21	$7.61\times 10^{-2}$	43.11	$8.56\times 10^{-3}$
A4		12.75	0.286	112.78	2.15	5.02	$9.42\times 10^{-2}$	34.14	$1.08\times 10^{-2}$
A1		8.76	0.369	88.96	1.65	5.00	$5.70\times 10^{-2}$	40.53	$8.39\times 10^{-3}$
A2	Great Vessels	12.75	0.012	376.15	3.97	4.68	$7.33\times 10^{-2}$	37.08	$9.68\times 10^{-3}$
A3	(Luminal Surface)	8.02	0.674	14.78	0.60	5.28	$5.42\times 10^{-2}$	41.48	$8.15\times 10^{-3}$
A4		14.70	0.319	152.86	2.50	4.75	$7.34\times 10^{-2}$	34.59	$9.99\times 10^{-3}$

**Table 4:** The mean and the maximum value of the fit error in percentage. Error values for fit of each part of the heart (*n* = 6) of each heart (*n* = 4) measurements are calculated as the mean value across all frequency points. The mean error is the mean across all parts of each of the hearts. The maximum error is the maximum across all parts of each of the hearts.

	Mean Error (%)	Maximum Error (%)
Relative permittivity	0.35	0.51
Conductivity	0.61	0.75

## Data Availability

All the data from this experiment is available at https://doi.org/10.5281/zenodo.3755616 (accessed on 15 March 2021).

## Abbreviations

The following abbreviations are used in this manuscript:

radiofrequency	RF
microwave	MW
electromagnetic	EM
atrial fibrillation	AF
vector network analyzer	VNA
E8362B PNA	VNA1
5063A ENA	VNA2
deionized water	DIW
sodium chloride	NaCl
animal 1–animal 4	A1–A4
standard deviation	SD
